# Development of an efficient autoinducible expression system by promoter engineering in *Bacillus subtilis*

**DOI:** 10.1186/s12934-016-0464-0

**Published:** 2016-04-25

**Authors:** Chengran Guan, Wenjing Cui, Jintao Cheng, Li Zhou, Zhongmei Liu, Zhemin Zhou

**Affiliations:** School of Biotechnology, Key Laboratory of Industrial Biotechnology (Ministry of Education), Jiangnan University, 1800 Lihu Avenue, Wuxi, 214122 Jiangsu China

**Keywords:** *Bacillus subtilis*, Promoter engineering, Expression system, Auto-inducible, High-cell-density fermentation

## Abstract

**Background:**

*Bacillus subtilis*, a Gram-positive organism, has been developed to be an attractive expression platform to produce both secreted and cytoplasmic proteins owing to its prominent biological characteristics. We previously developed an auto-inducible expression system containing the *srfA* promoter (P_*srfA*_) which was activated by the signal molecules acting in the quorum-sensing pathway for competence. The P_*srfA*_ promoter exhibited the unique property of inducer-free activity that is closely correlated with cell density.

**Results:**

To improve the P_*srfA*_-mediated expression system to the high-cell-density fermentation for industrial production in the *B. subtilis* mutant strain that is unable to sporulate, a spore mutant strain BSG1682 was developed, and the P_*srfA*_ promoter was enhanced by promoter engineering. Using green fluorescent protein (GFP) as the reporter, higher fluorescent intensity was observed in BSG1682 with expression from either plasmid or chromosome than that of the wild type *B. subtilis* 168. Thereafter, the P_*srfA*_ was engineered, yielding a library of P_*srfA*_ derivatives varied in the strength of GFP expression. The P23 promoter exhibited the best performance, almost twofold stronger than that of P_*srfA*_. Two heterologous proteins, aminopeptidase (AP) and nattokinase (NK), were successfully overproduced under the control of P23 in BSG1682. Finally, the capacity of the expression system was demonstrated in batch fermentation in a 5-L fermenter.

**Conclusions:**

The expression system demonstrates prominence in the activity of the auto-inducible promoter. Desired proteins could be highly and stably produced by integrating the corresponding genes downstream of the promoter on the plasmid or the chromosome in strain BSG1682. The expression system is conducive to the industrial production of pharmaceuticals and heterologous proteins in high-cell-density fermentation in BSG1682.

## Background

*Bacillus subtilis*, a Gram-positive soil bacterium, has been used for a long time to produce abundant industrial proteins. This bacterium has been used as an standard host strain because of its prominent characteristics, including its status as generally recognized as safe (GRAS), high-cell-density growth, well-characterized mechanisms of protein secretion, well-established methods for genetic manipulation, and applicability for large scale industrial production [1, 2]. Over the years, many enzymes and chemicals of clinical or industrial interest have been expressed in *B. subtilis*, such as pyridoxine, endoglucanase, alkaline protease, and surfactin [3–6].

One of the major strategies for expressing heterologous and homologous proteins is to construct expression systems with strong promoters. For many years, expression systems with strong promoters have been developed in *B. subtilis*. Most systems contain inducible promoters, such as P_*spac*_ induced by IPTG, P_*xylA*_ induced by xylose [7, 8]. One good example is the subtilin-regulated expression system (SURE) where the high level gene expression is strictly controlled by subtilin [9]. There are also inducer-free expression systems based on growth phase- or stress-specific promoters, such as the promoter of the *rpsF* operon or the promoter of the *pst* operon [10, 11]. Recently, a self-induction system has been developed in strain TQ356 [12]. However, current priority is given to develop novel, less technically challenging bacterial gene expression systems to accommodate the growing number of heterologous proteins to be expressed in *B*. *subtilis*.

The most important element of an expression system is the promoter. Compared with discovering novel promoters from the chromosome, engineering existing promoters is more convenient. One commonly used approach is to change the core regions of the promoter. For instance, derivatives of the *groES*-*groEL* promoter were generated by optimizing nucleotides of the conserved regions, including the UP element, −35, −16, −10 and +1 regions. Combination of these changes into one promoter increased the amount of recombinant proteins accumulated intracellularly up to about 30 % of the total cellular protein [13]. Similar modifications were carried out with the core region of the *aprE* promoter and the *cry3Aa* promoter, resulting in improved transcription activity [14, 15]. The other strategy of promoter engineering is to arrange at least two promoters upstream of the structural gene: a tandem repetitive sequences could consisted of the same promoters or different one. In *E. coli*, transcription strength of the MCP*tacs* promoter clusters shows a stepwise enhancement with the increase of the number of tandem repeats until reaching the critical value of five [16]. Additionally, TSaGT productivity was elevated several folds by the sequential alignment of the *HpaII* promoter with the *blma* promoter or the *amyR2* promoter in *B. subtilis* [17].

Previously, we constructed an auto-regulatory gene expression system with the *srfA* promoter (P_*srfA*_), which was triggered by signal molecules acting in the quorum sensing pathway for competence [18]. The P_*srfA*_ was shown to be strong and self-inducing. In order to adapt to large scale production, the mutant strain BSG1682 which was unable to sporulate, was constructed, and the P_*srfA*_-mediated expression of GFP was increased in BSG1682 via plasmid or chromosomal integration. Next, the strength of P_*srfA*_ was improved by promoter engineering, and the mutant labeled P23 was shown to be most effective in activating the expression of GFP. Two heterologous proteins, aminopeptidase (AP) and nattokinase (NK) were successfully produced under the control of P23 in BSG1682. The capacity of the expression system was demonstrated in a 5-L fermenter. These results suggested that the novel expression system could be used for industrial scale production of heterologous proteins in *B. subtilis*.

## Results

### Features of the native the P_*srfA*_ promoter

Although diverse homologous and heterologous proteins have been successfully overproduced in *B. subtilis* under the control of inducible promoters, a noticeable basal expression was observed. Moreover, the high cost of inducers added into media during fermentation in large scale limits the industrial utilization of these protein expression systems [7].

The *srf* operon, required for the production of the lipopeptide antibiotic surfactin, competence development, and efficient sporulation, is activated by the mechanism of quorum sensing in *B. subtilis* [19]. As cells grow, two signaling peptides ComX and CSF were accumulated to the threshold level to activate the signal transduction system that is composed of the two-component regulatory proteins ComP and ComA. Finally, the phosphorylated ComA binds to the promoter of the *srf* operon (P_*srfA*_) to initiate the transcription of downstream genes [20, 21]. Previously, we characterized the P_*srfA*_ by constructing the plasmid pBSG03 with GFP as the reporter protein. P_*srfA*_ was prominent for its strength and autoinducibility. During the lag phase and the early exponential phase, there was little fluorescence detected. Fluorescence began to emerge at the mid-exponential phase; the intensity sharply increased to the peak value during the transition to stationary phase, and remained constant during the followed stationary phase [18]. In this work, to apply the auto-inducible expression system to high-cell-density fermentation for industrial production, a nonsporulating strain BSG1682 was constructed and the transcription activity was enhanced.

### Construction of the spore mutant strain and evaluation of GFP expression

To avoid sporulation during expression and the risk of spores persisting in the fermentor, the gene *σ*^*F*^ was deleted from the chromosome of *B. subtilis* 168, making the nonsporulating strain BSG1682.

**Fig. 1:**
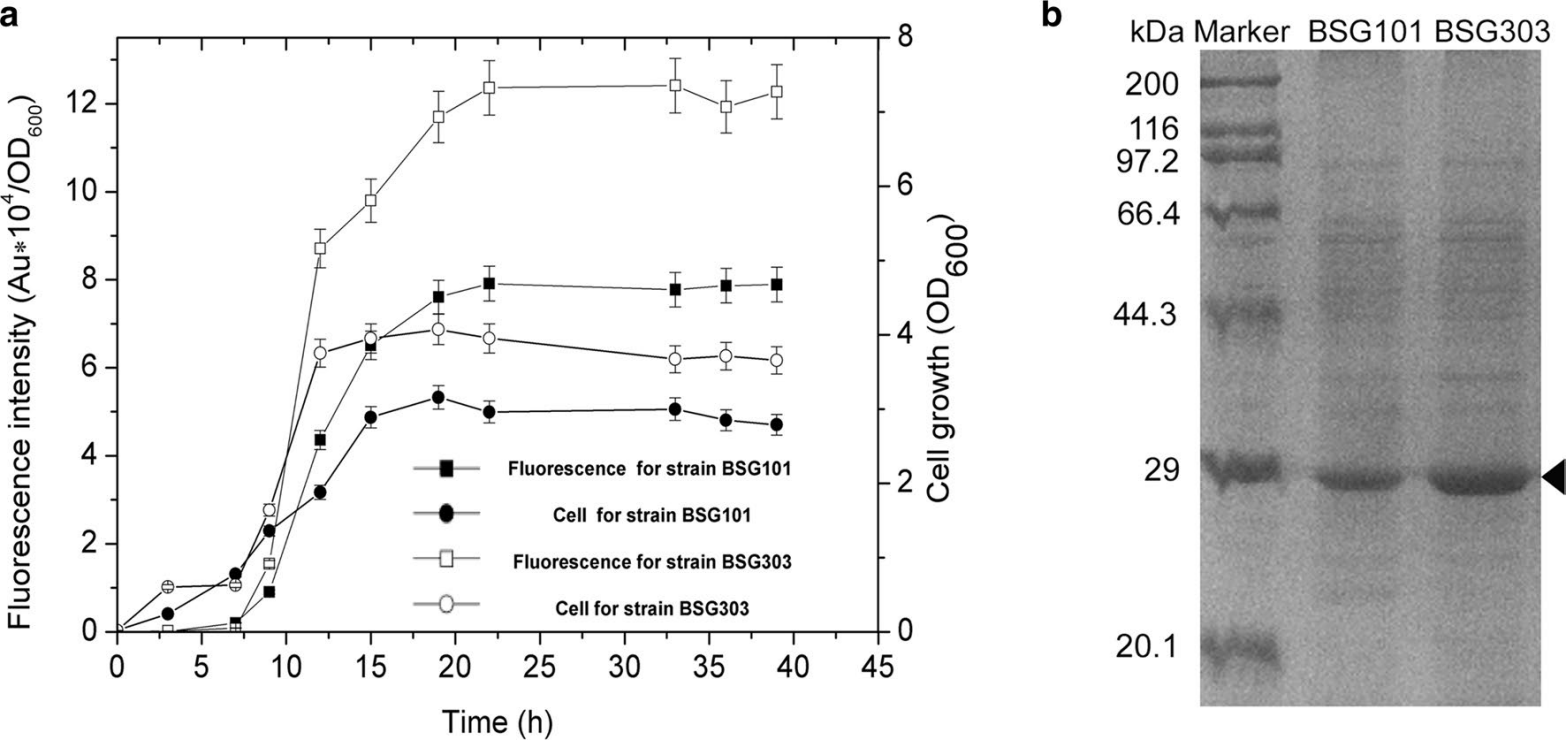
The expression level and pattern of GFP were measured in BSG101 and BSG303 (**a**) and the expression level was analyzed by the SDS-PAGE (**b**). The spore-deficient BSG1682 was obtained by deleting the *σ* ^*F*^ which was critical for all spore development in *B. subtilis* 168. To evaluate strain BSG1682 for the GFP expression, plasmid pBSG03 was transformed into BSG1682 and *B. subtilis* 168 yielding strains BSG303 and BSG101 respectively. These two strains were cultivated in the same procedure and periodically sampled

To assess the strain BSG1682 for protein production, expression of GFP in BSG1682 was compared with that of the wild-type background *B. subtilis* 168 which was demonstrated to be the optimal host for the P_*srfA*_ system [18]. The plasmid pBSG03 was transformed into BSG1682 yielding the recombinant strain BSG303, and then together with BSG101 were treated with the same procedure. The growth rate and cell density of BSG303 were superior to the strain BSG101 based on cell growth curve (Fig. 1a). Fluorescence intensity measured in BSG303 was 60 % higher than that of BSG101. The GFP expression level could reach up to 7.8 and 11.7 % of total intracellular proteins in BSG101 and in BSG303, respectively, which was confirmed by SDS-PAGE analysis (Fig. 1a, b). These results indicated that the spore mutant not only had advantage in cell growth but also in the production of GFP.

### Construction of an integrative expression system

**Fig. 2:**
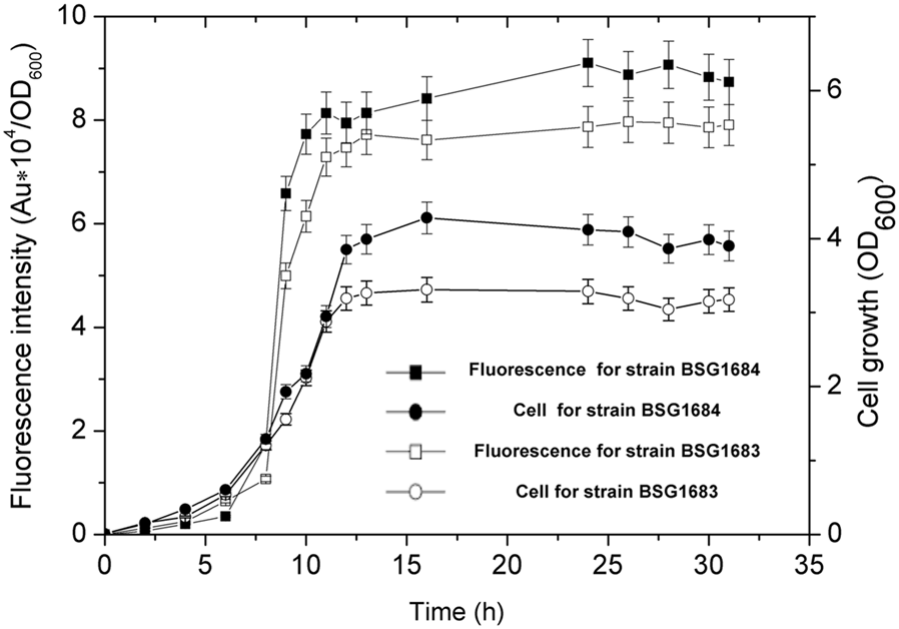
Identification of the integration expression level and pattern of GFP in BSG1683 and BSG1684. The fragment containing the P_*srfA*_ promoter and the *gfp* gene was separately integrated into the chromosome of *B. subtilis* 168 and BSG1682, and the resulting recombinant strains BSG1683 and BSG1684 were cultivated for GFP expression

To perform chromosomal integration, plasmid pAX01was used for ectopic integration at the *amyE* locus of *B. subtilis*, enabling expression from a stable chromosomal site as a single copy. The fragment containing P_*srfA*_ and the *gfp* gene was cloned into pAX01 to yield plasmid pAX01-GFP. The recombinant plasmid was transformed into strain *B. subtilis* 168 and BSG1682, yielding the chromosomally integrated derivatives of BSG1683 and BSG1684, respectively. These two strains were cultivated by the standard procedure to express GFP. Cell mass and fluorescence intensity measured in BSG1684 were obviously higher than those in BSG1683 (Fig. 2). This result further demonstrated that the spore mutant strain was better for the expression system than the wild-type background of *B. subtilis* 168.

### Engineering the promoter core regions to improve expression

**Fig. 3:**
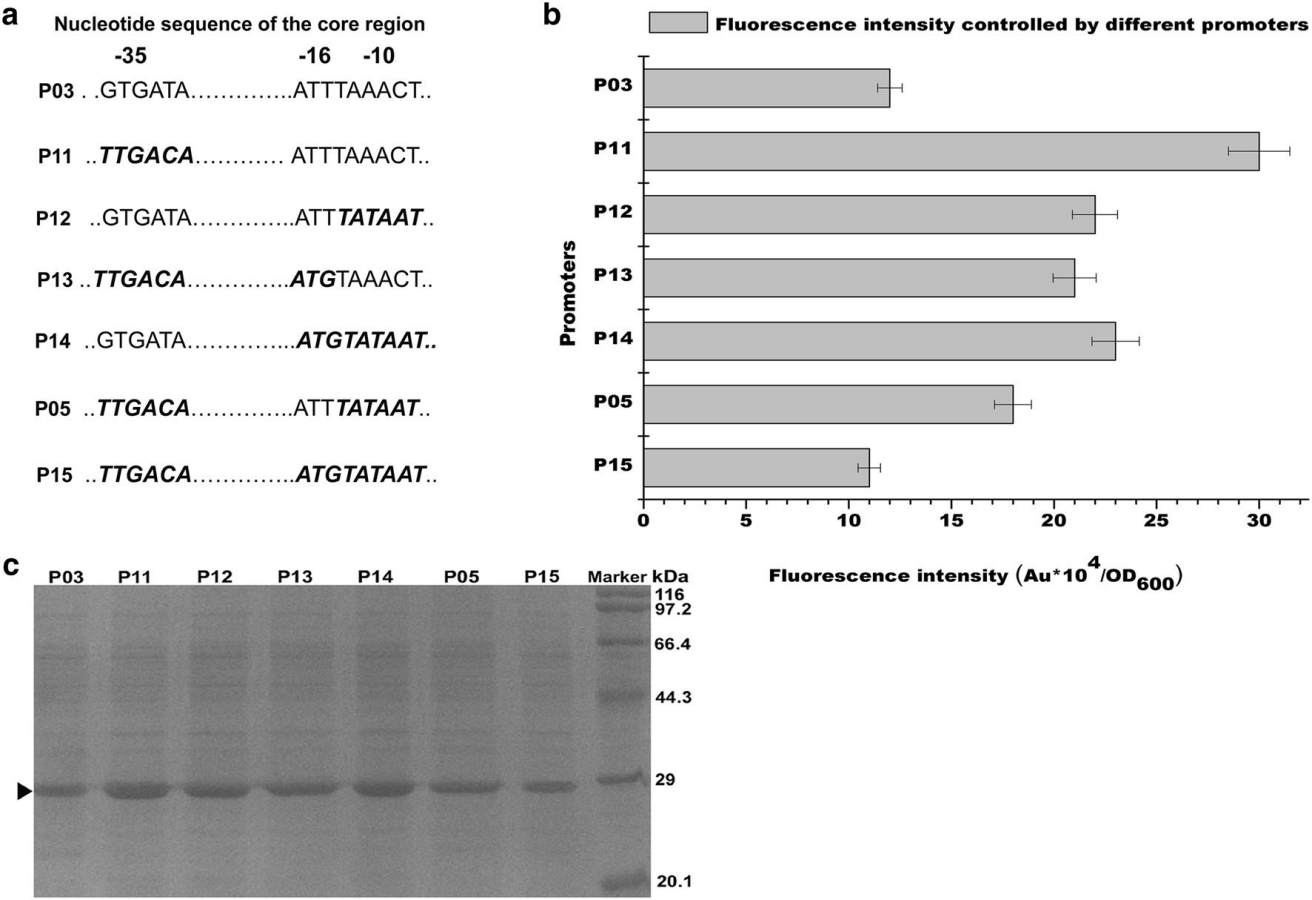
The sequences of the core regions in the relative derivatives of the P_*srfA*_ promoter (**a**). The core regions of the P_*srfA*_ promoter (labeled as P03 in the figure) were changed individually or in combination to the corresponding consensus sequence. The mutant sequences compared to the wild-type sequences of the P03 promoter were represented in bold italic. The fluorescence intensity controlled by the derivatives (**b**) and SDS-PAGE analysis of the GFP expression (**c**). The band corresponding to GFP was marked

To enhance transcription, the −10, −16, and −35 regions of P_*srfA*_ on pBSG03 were changed into the corresponding consensus sequence separately or in combination (Fig. 3a). Several plasmids containing the mutant P_*srfA*_ were obtained and transformed into BSG1682 to be treated by the standard procedure. Compared with the wild-type P_*srfA*_ promoter (P03), all of the mutant promoters except the P15 promoter exhibited degrees of increase in promoter strength (Fig. 3b). A promoter with the consensus −35 hexamer (P11) showed best performance among all and which was 1.5-fold stronger than the P03 promoter. The GFP expression level controlled by the P11 promoter reached up to 26.4 % of total intracellular proteins (Fig. 3c). The activities of promoters with mutations in −10 region (P12), concurrently mutations in −16 region (P13 and P14) or −35 region (P05) also showed higher expression than the wild-type promoter. However, when combining all of these improvements into a single promoter (P15), the expression of GFP was less than that of the wide type promoter (Fig. 3b). These findings were also confirmed by the SDS-PAGE (Fig. 3c).

### Development of dual promoters and mutations in the core region to enhance the protein expression

**Fig. 4:**
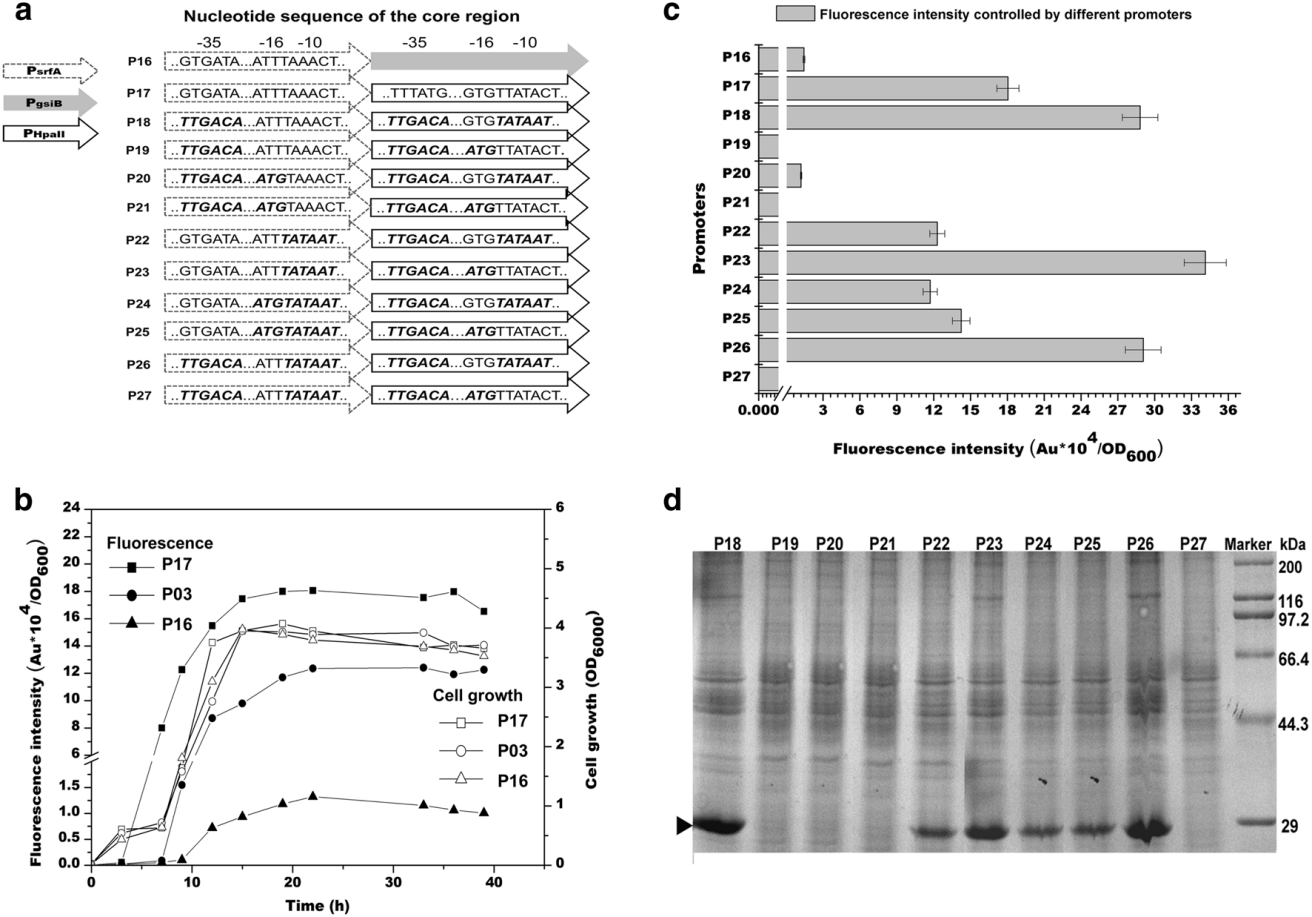
The core region of the two dual promoters and the derivatives of the P_*srfA*_-P_*HpaII*_ dual promoter (**a**). Two dual promoters were constructed to increase the expression and the core regions of P_*srfA*_-P_*HpaII*_ dual promoter (named as P17 in the figure) were changed individually or in combination to the corresponding consensus sequences yielding the mutants of the P18 promoter to the P27 promoter. The mutant sequences compared to the wild-type sequences of the P17 promoter were represented in *bold italic*. The expression patterns of the dual promoters were compared to that of the P_*srfA*_ promoter (**b**). The fluorescence intensity controlled by the derivatives of P_*srfA*_-P_*HpaII*_ (**c**) and SDS-PAGE analysis of GFP controlled by the corresponding promoters (**d**). The band corresponding to GFP was marked

Another strategy wildly used for developing stronger promoters is to use at least two promoters in tandem. Considering that the window of P_*srfA*_ activity was confined to the phase of exponential growth, two research ways are proposed to enhance the expression activity of the system. One is to extend the window for the target gene expression. Therefore, the *gsiB* promoter (P_*gsiB*_), recognized by *σ*^*B*^, was linked to the P_*srfA*_ promoter for gene expression on plasmid pBSG03, yielding pBSG16. The other is to enhance the promoter activity within the window. Thus, the P_*HpaII*_ promoter, recognized by *σ*^*A*^, was put immediately downstream of P_*srfA*_, yielding pBSG17 (Fig. 4a).

To evaluate the dual promoters for GFP expression, plasmid pBSG16 and pBSG17 were transformed into strain BSG1682, yielding recombinant strain BSG316 and BSG317, respectively. Though the P_*gsiB*_ promoter was typically used as a strong promoter for gene expression in *B. subtilis*, the GFP expression induced by the P_*srfA*_-P_*gsiB*_ dual promoter (P16) was only 12 % of that by the P03 promoter (Fig. 4a, c). The strength of the P_*srfA*_-P_*HpaII*_ dual promoter (P17) was higher than that of the P_*srfA*_. The GFP yield triggered by P_*srfA*_-P_*HpaII*_ was 0.5-fold higher than that of the P03 promoter (Fig. 4a, c).

To examine these data in detail, expression characteristics of the dual promoters were studied (Fig. 4b). For the wild-type P03 promoter, fluorescence was measured during the mid-exponential phase and the transition to stationary phase as previously performed. The P_*srfA*_-P_*HpaII*_ dual promoter (P17) was in agreement with our expectation that the combined promoter activity was enhanced during the given time window. However, the expression window could not be extended by the *σ*^*B*^-recognized P_*gsiB*_, and P_*srfA*_-P_*gsiB*_ (P16) showed weaker strength than the P03 promoter across the whole time. The unexpected result of GFP expression by the dual promoter P_*srfA*_-P_*gsiB*_ was in consistent with the former report that the synergistic effect of the double promoters was observed only when the *σ*^*B*^-promoter was located upstream the *σ*^*A*^-promoter [22]. However, to test whether the result was caused by the arrangement of the two promoters or the property of the target protein, further experiments are warranted.

As the P_*srfA*_-P_*HpaII*_ dual promoter (P17) exhibited better performance in GFP expression, the −35, −10 and −16 regions of the dual promoters were changed separately or in combination to the consensus sequence as performed above (Fig. 4a). A set of plasmids containing dual promoters with mutated regions were constructed and transformed into BSG1682 to evaluate promoter activity (Fig. 4a, c). When the positive mutations evaluated in the P03 promoter were randomly combined into the dual promoter, some dual promoter derivatives showed unexpectedly low expression compared to their equivalents in the P_*srfA*_ promoter, such as P19, P20, P21, and P27. Among all of the promoter mutants, the P23 promoter showed the strongest GFP expression, which was 0.9-fold higher than that of the original dual promoter P17 (Fig. 4c). Though the activity of the P11 promoter was higher than the P14 promoter (Fig. 3), the strength of the P23 promoter (the derivative of the P14 promoter) was 20 % stronger than that of the P18 promoter (the derivative of the P11 promoter). These findings were in agreement with the analysis of SDS-PAGE (Fig. 4d).

### Heterologous proteins expression

**Fig. 5:**
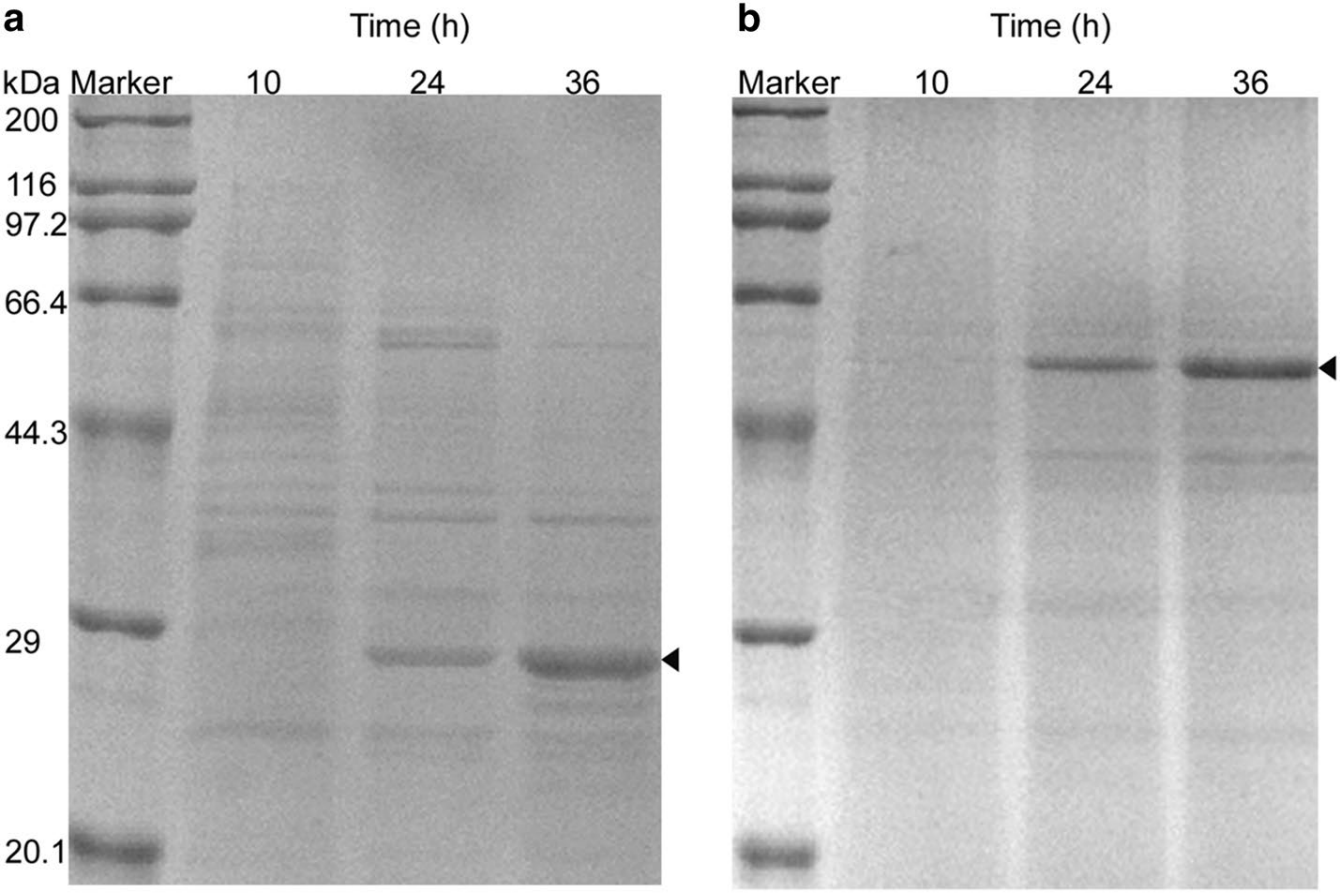
Overproduction of NK (**a**) and AP (**b**) using the P23-mediated expression system. The heterologous proteins NK of and AP were inserted downstream of the P23 promoter, resulting in relative plasmids pBSG28 and pBSG29. The corresponding recombinant strains BSG328 and BSG329 were cultivated and periodically sampled. The bands indicating to the target proteins were marked

As the P23 promoter was the strongest promoter in our study, the corresponding plasmid pBSG23 was used for the heterologous proteins expression experiments to evaluate the applicability of the expression system. One heterologous protein was nattokinase (NK), isolated from *B. subtilis natto*, consisted of 353 amino acids and used for preventing hypertension and cardiovascular diseases because of its strong fibrinolytic activity. The other heterologous protein was aminopeptidase (AP), isolated from *B. subtilis* Zj016, classified to the M28 Family, and catalyzed the cleavage of the N-terminal amino acid residues from peptides and proteins. The fragment containing the signal peptide of *Bpr* and the coding sequence (CDS) of NK and the gene sequence carrying the intrinsic signal peptide and the CDS of AP were cloned and separately inserted downstream of the P23 promoter on pBSG23 to replace the *gfp* gene, yielding pBSG28 and pBSG29, respectively. These two recombinant plasmids were transformed into BSG1682 and were treated in the same manner as described above. These two heterologous proteins were successfully overexpressed (Fig. 5). The fibrinolytic activity of NK measured from the supernatant was as high as 93.6 Fu/mL. The activity of AP was 69.8 U/mL which was higher than that of the P_*srfA*_ promoter and the P_*HpaII*_ promoter [18, 23].

### Evaluation of the expression system in a 5-L bioreactor

**Fig. 6:**
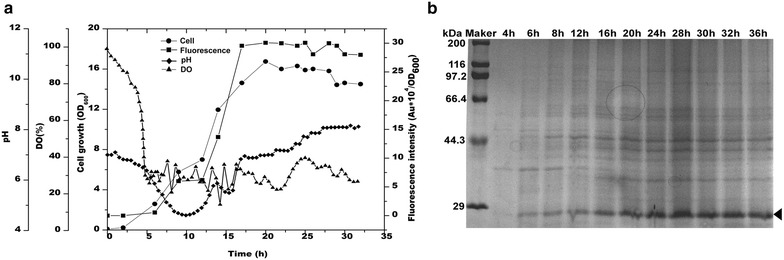
Batch fermentation of GFP in the recombinant strain BSG323 (**a**). SDS-PAGE analysis of the GFP expression (**b**). The samples were periodically sampled and the band corresponding to GFP is marked

Scaling up from the above results of cultivation in the shaken flask, the industrial scale capacity of the expression system was determined by performing fermentation experiments in a 5-L fermentor (Fig. 6a). The cells grew fast and the biomass determined by OD_600_ was 3.2-folds higher than that of the flask. Additionally, the fluorescence was significantly increased at the turning point of pH, as well as at the exhaustion of glucose. The fluorescence intensity was 85 % of that detected in the flask and the GFP expression level reached 28.4 % of total intracellular proteins. The expression was also verified at the protein level by SDS-PAGE analysis (Fig. 6b).

## Discussion

*Bacillus subtilis* has garnered increasing attention as a promising platform for the industrial production of various heterologous proteins via diverse expression systems. The promoter is one of the key elements for a successful expression system. In our previous work, the P_*srfA*_ promoter was shown to be strong and autoinducible in heterologous gene expression in *B. subtilis* [18]. To apply the expression system to the industrial scale production, a spore-deficient host BSG1682 was constructed by deleting the gene of *σ*^*F*^ in that a *σ*^*F*^-deleted strain was reported to form less spores [17, 19]. Compared with our previous work, BSG1682 was demonstrated to be the most optimal chassis for the P_*srfA*_-mediated expression with improved expression of GFP. One hypothesis for the improvement in BSG1682 was that the energy loss and chemical energy consumption required for sporulation were reduced. Additionally, the expression period was prolonged [12, 24], which might contribute to high expression. Additionally, considering on the closely related processes of competence and sporulation in *B. subtilis* [19, 25, 26], the deletion of *σ*^*F*^ might cause certain proteins or molecules to work on the regulation of the *srf* operon to promote the P_*srfA*_ promoter and increase the yield.

Chromosomal integration of the gene of interest is a powerful alternative to deal with problems of expression instability in plasmid-based systems in long periods of industrial scale fermentation. Integration is especially suitable for metabolic engineering where a constant gene copy number is valuable in synthetic control circuits while temporal variation in copy number could be problematic, leading to unintended responses [27]. In this study, the integrative expression strain BSG1684 was a good tool for chromosomal expression because of its genetic stability. Though the GFP expression of BSG1684 was lower than that of the plasmid-based BSG303, expression could be improved by CIChE-engineering multiple copies of expression elements into the chromosome [28].

As one of the most efficient and prevalent strategies, promoter engineering has been widely used to tune the expression level of the targeted genes. It is used both in protein overexpression and in tunable synthetic genetic circuit [29, 30]. One of the widely used methods of promoter engineering is to change the promoter core region into consensus sequence [31]. It has been reported that there are four elements that are closely correlated with promoter activity, −10 region, −35 region, the UP element, and the −16 sequence [32, 33]. By changing the core elements of P_*srfA*_ into the consensus recognition sequence of the *σ*^*A*^-dependent promoters in *B. subtilis* [32, 34, 35], the P11 promoter showed the strongest activity. This result was in accordance with previous findings that the promoter activities of P_*aprN*_ and P_*groES*-*groEL*_ were improved by modifying the −35 region [13, 31]. However, combining all of the mutations with improvements into one single promoter (P15), the activity was decreased, which was different from the effects obtained from the similar modification on P_*aprN*_ and P_*groES*-*groEL*_. These results suggested that modification of the promoter core region was not a general method to enhance the promoter strength, and other aspects should be simultaneously taken into account, including the property and structure of the promoter and the target protein.

The capacity of the expression system used for high cell density fermentation was explored in a fermenter. Contrary to the higher biomass, the fluorescence intensity was lower than that of the flask experiment. One possible explanation for this data was that the nutrient was more used for cell growth than for the GFP production. Another possibility was that the acidic conditions caused by the consumption of glucose might narrow the time window of activation of the promoter. The *srf* expression was demonstrated to be apparent pH-dependent induction as raising the pH to near neutrality resulted in dramatic increases in *srf* expression [36]. Therefore, to improve production from the expression system further, it is worth attempting to systematically optimize the fermentation process, including changing the feeding strategies and keeping the pH higher than six during the acidic phase of fermentation.

As the rapid and versatile cloning method of sequence and ligation-independent cloning (SLIC) has been widely used, it is convenient to construct a library or convert an existing library into a different background [37]. Assisted with SLIC, the mutant promoters with various activities constructed in this study could be used for specific applications, including metabolic engineering and control analysis, which require a large number of promoters with strengths that vary in only slight increments to cover a specific window around the wild-type expression levels of the studied gene.

## Conclusions

*Bacillus subtilis* has been used as a promising biofactory to overexpress heterologous genes owing to its prominent characteristics. To enrich the library of expression tool, an optimized expression system based on the P23 promoter in the strain BSG1682 was developed with improved expression of target genes. The P23 promoter could also be used for expression from chromosomal integration expression for its strength and stable activation. The derivatives of the P_*srfA*_ promoter might be used in metabolic engineering for tuning gene expression because of the varied strength and stable expression. In summary, the novel expression system could be applied for industrial production of pharmaceuticals and heterologous proteins in BSG1682.

## Methods

### Strains, plasmids and growth conditions

Bacterial strains and plasmids used in this study are listed in Table 1. Plasmid pAX01 was purchased from Biovector NTCC (Beijing, China). *Escherichiacoli* JM109 was used as the host for gene cloning. *Bacillus subtilis* 168 and BSG1682 were used for gene expression and integration. All strains were cultured in the appropriate medium with aeration at 37 °C. When appropriate, *B. subtilis* growth medium was supplemented with kanamycin (5 μg/mL) and *E. coli* growth media was supplemented with ampicillin (10 μg/mL). Cell densities were determined by measuring OD_600_ with a UV-1800/PC spectrophotometer (Shanghai, MAPADA Instrument Co., Ltd., China).

**Table 1:** Strains and plasmids used in this study

Strains and plasmids	Relevant properties	Reference
Strains		
*E. coli* JM109	*recA*1, *endA*1, *gyrA*96, *thi*-1, *hsd*R17(r_k-_m_k-_), e14^−^(*mcrA* ^−^), *supE*44, relA1, Δ(lac-proAB)/F’ [traD36, proAB^+^, lacIq, lacZΔM15]	
*B. subtilis* 168	trpC2	This work
BSG1682	(*B. subtilis* 168) Δ*σ* ^*F*^	This work
BSG1683	The chromosome of *B. subtilis* 168 integrated with the fragment containing the P_*srfA*_ and the *gfp* gene	This work
BSG1684	The chromosome of BSG1682 integrated with the fragment containing the P_*srfA*_ and the *gfp* gene	This work
BSG101	*B. subtilis* 168, pBSG03 (P_*srfA*_-GFP)	[18]
BSG303	BSG1682, pBSG03 (P_*srfA*_-GFP)	This work
BSG305	BSG1682, pBSG05 (^mut^P_*srfA*_-GFP)	This work
BSG3x	BSG1682, pBSGx (Px-GFP)	This work
BSG328	BSG1682, pBSG28 (P23-NK)	This work
BSG329	BSG1682, pBSG29 (P23-AP)	This work
Plasmids		
P7Z6	*zeo* ^r^, *bla* ^r^, Cre/lox	[40]
pUC19	pUC origin, P_*lac*_, *Ap* ^r^	Takara
pAX01	P_*xylA*_, *erm* ^r^, *bla* ^r^, *lacA*	[45]
pAX01-GFP	pAX01 containing P_*srfA*_ and GFP	This work
pET-24a-*nk*	pET-24a containing signal peptide of *Bpr* and the CDS of NK	In our lab
pBSG03	GFP ligated downstream of P_*srfA*_	[18]
pBSG04	pBSG03 with P_*srfA*_ replaced by P_*HpaII*_	[18]
pBSG05	pBSG03 with ^mut^P_*srfA*_ (^mut^P_*srfA*_ was referred as P05 in this work)	[18]
pBSG06	pBSG05 with GFP replaced by the sequence containing the intrinsic signal peptide and the CDS of AP	[18]
pBSG3x	The derivatives of pBSG03 containing parallel promoter of Px (from P11 to P27)	This work
pBSG28	Derivative of pBSG23 with GFP replaced by the fragment containing the signal peptide of *Bpr* and the CDS of NK	This work
pBSG29	Derivative of pBSG23 with GFP replaced by the sequence loading the signal peptide and the CDS of AP	This work

### Recombinant DNA techniques

Plasmid construction was performed in *E. coli* and DNA extraction was performed by following a standard procedure as previously described [38]. Recombinant plasmids were transformed into *B. subtilis* as previously described [23]. Enzymes were obtained from TOYOBO (Osaka, Japan), TaKaRa (Dalian, China), or NEW ENGLAND BioLabs (Beijing, China) and were used according to the manufacturers’ protocols. The primers used in this study are listed in Table 2. PCR was performed using KOD DNA polymerase (Osaka, Japan). All of the recombinant plasmids constructed in this work were confirmed by DNA sequencing (Shanghai Sangon Biotech Co., Ltd., China).

**Table 2:** Oligonucleotides used in this study

Primer	Nucleotide sequence of primer^a, b^
P1F	TTTGTT **ACTTCCTAGAATATATATTATGTAAACT** CTTGATATGGCTTTTTATATGTG
P1R	GCAGA**AGTTTACATAATATATATTCTAGGAAGT**TCAGTCCTGCTCCTCGGCCACGAAG
P2F	AGTACTCGCTGAAAGTCCTGTTGCTGC
P2R	ATCAAG**AGTTTACATAATATATATTCTAGGAAGT** AACAAATCTCCTTAATTACAAAGCG
P3F	ACTGA **ACTTCCTAGAATATATATTATGTAAACT** TCTGCAGTGCAGGCTAGCTTTTTTGTGC
P3R	CCGACGAACAAACCTGCCAGAAGCCC
P11F	ACTTTTCACCCATTTTTCGG***TTGACA***AAAACATTTTTTTCATTTAAACTGAACGGTA
P11R	TTTAAATGAAAAAAATGTTTT***TGTCAA***CCGAAAAATGGGTGAAAAGTTTCATGCGGG
P12F	ATAAAAACATTTTTTTCATT***TATAAT***GAACGGTAGAAAGATAAAAAATATTGAAA
P12R	TTTTATCTTTCTACCGTTC***ATTATA***AATGAAAAAAATGTTTTTATCACCGAAAAA
P13F	ACTTTTCACCCATTTTTCGG***TTGACA***AAAACATTTTTTTC***ATG***TAAACTGAACGGTA
P13R	TTTA***CAT***GAAAAAAATGTTT***TGTCAA***CCGAAAAATGGGTGAAAAGTTTTCATGCGGG
P14F	ATAAAAACATTTTTTTC***ATGTATAAT***GAACGGTAGAAAGATAAAAAATATTGAAA
P14R	TTTTATCTTTCTACCGTTC***ATTATACAT***GAAAAAAATGTTTTTATCACCGAAAAA
P15F	ACTTTTCACCCATTTTTCGG***TTGACA***AAAACATTTTTTTC***ATGTATAAT***GAACGGTAGAAAGATAAAAAATATTGAAA
P15R	TTTTATCTTTCTACCGTTC***ATTATACATG***AAAAAAATGTTTT***TGTCAA***CCGAAAAATGGGTGAAAAGTTTCATGCGGG
P16F	TGTTAGTTCATAAGAATTAAAATTTATGAATATAAAGTATAGTGTGTTATACTTGCTGATATGAGAAAATGCGTTGCACATGGGATAAGAAA
P16R	GCATTTTCTCTTTCTTATCCATATCAGCAAGTATAACACACTATACTTTATATTCATAAATTTTAATTCTTATGAACTAACAGCCG
P17F	AAGCTGATATGGATAAGAAAGTTTAAAAGAATTGTGAGCGGGAATACAACAACCAACACCAATTAAAGGAGGAAGACAATGATGAGTAAAGGAGAAGAACTTTTCACTGGAG
P17R	TTCTTCTCCTTTACTCATCATTGTCTTCCTCCTTTAATTGGTGTTGGTTGTTGTATTCCCGCTCACAATTCTTTTAAACTTTCTTATCCATATCAGCTTTTAATTCTTATGAACTAACAGCCG
P18F	TGTTAGTTCATAAGAATTAAAA***TTGACA***AATATAAAGTATAGTGTGT***TATAAT***GCTGATATGGATAAGAAAGAGAAAATGC
P18R	TTTCTTATCCATATCAGC***ATTAT***AACACACTATACTTTATATT***TGTCAA***TTTAATTCTTATGAACTAACAGCCGAAATAG
P19F	TGTTAGTTCATAAGAATTAAAA***TTGACA***AATATAAAGTATAGTATGTTATACTTGCTGAT***ATG***GATAAGAAA
P19R	CCATATCAGCAAGTATAACATACTATACTTTATATT***TGTCAA***TTTTAATTCTTATGAACT
P28F	CTGCTTATAAAGATTAGGGGAGGTATGACAATGATGAGGAAAAAAACGAAAAACAG
P28R	CCGCACAGATGCGTAAGGAGAAAATACCGCTTATTGTGCAGCTGCTTGTACG
P29F	CTGCTTATAAAGATTAGGGGAGGTATGACAATGATGAAAAAGCTTTTGACTGTC
P29R	CCGCACAGATGCGTAAGGAGAAAATACCGCTTATTTGATATCTTCAAAAATG
P30F	CTTCAATAATATTGAAAAAGGAAGAGTGCGGCCGCATCGACAAAAATGTCATGAA
P30R	CAAAGGGGGAAATGGGATCCGAGCTCCCGCGGTTATTTGTATAGTTCATCCATG

^a^Homologous sequences were underlined; dif_*B. subtilis*_ sequence was shown in bold and the mutant sequences were shown in italic bold

^b^The nucleotide sequence of primers P20R/F, P22R/F, P24R/F and P26R/F were the same as P18R/F; the nucleotide sequence of primers P21R/F, P23R/F, P25R/F and P27R/F were the same as P19R/F

### Construction of markerless deletion mutant strain

The markerless deletions of genes on the chromosome of *B. subtilis* 168 were performed as previously described, with certain modification [39, 40]. The sequences of *B. subtilisdif* site (dif _*B. subtilis*_: ACTTCCTAGAATATATATTATGTAAACT) was used in this study.

To delete the gene *σ*^*F*^ from the *B. subtilis* 168 chromosome, it was necessary to provide three fragments. The *zeo*-sequence, the zeocin resistance gene *zeo*, was amplified from plasmid p7Z6 using primers P1F and P1R. These primers incorporated a 3′ region of homology flanking the *zeo* gene and a 5′ tail that included a 5-bp sequence homologous to the chromosomal regions flanking gene *σ*^*F*^ and a 28-bp dif _*B. subtilis*_. The up-sequence (consisting of approximate 1-kb homologous fragment upstream of *σ*^*F*^, the dif _*B. subtilis*_ site, and 6-bp homologous with 5′ ends of the gene *zeo*), was cloned from *B. subtilis* 168 chromosome with primers P2F and P2R. The down-sequence (consisting of 5-bp homologous with 3′ ends of the gene *zeo*, the dif _*B. subtilis*_, and approximate 1-kb homologous fragment downstream of gene *σ*^*F*^) was amplified with primers P3F and P3R in the same manner as employed for the up-sequence. These three fragments were fused together in a second joining PCR reaction. The resulting 2.8-kb fragment was sequenced, and transformed into the competent *B. subtilis* 168. Integrant were selected on LB agar containing 25 μg/mL zeocin. Subculturing in the LB broth in the absence of antibiotics for approximately 36 h produced zeocin-sensitive recombinant clones, identified by replica plating onto agar with and without the selective antibiotic. Finally, strain BSG1682 (Δ*σ*^*F*^) was analyzed by PCR using primers P2F and P3R and sequenced to confirm the deletion of *σ*^*F*^.

### Plasmids constructions

To construct the plasmids with mutations in the core sequence of P_*srfA*_, plasmid pBSG03 was used as the template by means of a megaprimer PCR with a whole plasmid protocol [41]. The plasmid containing P_*srfA*_ derivatives were gained with the corresponding primers (listed in Table 2). The core fragment of P_*gsiB*_ and P_*HpaII*_ were synthesized into primers (P16F/P16R and P17F/P17R) and separately fused with P_*srfA*_ on plasmid pBSG03, yielding corresponding plasmids pBSG16 and pBSG17. The derivatives of pBSG17 (from pBSG18 to pBSG27 containing the relative promoters P18 to P27) with mutations in the core sequence of the promoters were obtained with the corresponding primers.

To construct plasmids for heterologous proteins expression, gene *nk* and *ap* were separately cloned from pET-24a-*nk* and pBSG06 with primers P28F/R and P29F/R, resulting plasmids of pBSG28 and pBSG29.

The integrative strain was generated in two steps. First, the gene fragment containing P_*srfA*_ and the *gfp* gene was cloned from pBSG03 with primers P30F and P30R, and the PCR products were genetically fused with plasmid pAX01, yielding plasmid pAX01-GFP. Second, pAX01-GFP was transformed into *B. subtilis* 168 and BSG1682, yielding strain BSG1683 and BSG1684, respectively.

### Cultivation of recombinant strain for expression of GFP

A single colony of the appropriate *B. subtilis* strain selected from an LB agar plate was inoculated into 5 mL of LB liquid medium and cultivated overnight for 12 h. Next, 0.6 mL preculture was inoculated into 250-mL shake flasks containing 30 mL of LB liquid medium. The shake flasks were shaken at 200 rpm and periodically sampled. Cells were harvested by centrifugation, washed by PBS buffer (50 mM Tris–HCl, 100 mM NaCl, pH 7.5) three times, and suspended at an appropriate dilution ratio.

Measurement of fluorescent activity was conducted by Synergy™ H4 multimode microplate reader (BioTek Instruments, Inc., USA) with 200 μL diluent in a 96-well microlon ELISA plate. Fluorescence readings were taken from the bottom using a GFP-specific filter pair (excitation 495/9 nm, emission 525/9 nm, gain value 80). Determination of the GFP expression was calculated as the derivative of the fluorescence divided by the OD _600_ (dGFP/OD _600_) and the expression level was calculated as previously described [18, 42]. Growth was monitored by measuring absorbance at 600 nm. All data were averaged from three independent samples of the same time points.

### Heterologous protein expression and enzyme activity analysis

A fresh overnight culture of the appropriate recombinant strain containing plasmid pBSG28 and pBSG29 was inoculated into 250-mL shake flasks containing 30 mL LB liquid medium, cultivated for 2 days and periodically sampled. A cell-free supernatant was obtained by centrifugation (5 min, 10,000×*g*). AP activity measurements were performed as previously described [43]. One unit was defined as the amount of enzyme that released 1 μmol L^−1^*p*-nitroaniline min^−1^ at 37 °C (ε _405 nm_ = 9.98 L mmol^−1^cm^−1^). For NK activity, the reaction was performed as described and one unit of fibrinolytic (FU) activity was defined as the amount of enzyme required to produce an absorbance increase equal to 0.01 in 1 min at 275 nm [44]. The data shown represent mean values from assays performed in triplicate.

### Batch fermentation

The experiment was carried out in a 5-L fermenter (Winpact Bench-Top, Major Science Inc., New Taipei City, Taiwan) containing 2 L of LB medium. A single colony of BSG323 from an LB agar plate was inoculated in 5 ml LB medium and cultivated at 37 °C for at least 12 h with vigorous shaking. Afterward, 2 % of the pre-culture was transferred into 500-mL shake flasks with a working volume of 100 mL of LB liquid medium. After 8 h of cultivation with shaking, the entire culture and 42 g glucose were transferred into the fermenter. Dissolved oxygen (DO) was monitored using a polarographic DO sensor and was maintained above 30 % saturation by controlling both the inlet air and the agitation rate between 200 and 800 rpm. Foaming was controlled by the addition of an anti-foaming agent (a mixture of organic polyether dispersions, Sigma).

### Protein analysis and SDS-PAGE

For GFP, crude cell extracts were obtained according the following protocols. The OD_600_ of every sample was measured, and a certain volume corresponding to approximately the same OD _600_ was harvested and centrifuged. The pelleted cells were resuspended with PBS buffer and disrupted by sonication on ice using ultrasonic cell crusher noise isolating chamber (XinChen biotechnology Co., LTD, China). The crude cell extract was separated by centrifugation. Afterward, the amount of GFP, as well as AP and NK, were determined by SDS-PAGE according to standard procedures. Gels were subsequently stained with Coomassie brilliant blue R250. The gel image analysis was performed by Glyko Bandscan (Version 5.0).

## Abbreviations

P_*srfA*_: the promoter of the *srf* operon
GFP: green fluorescent protein
AP: aminopeptidase
NK: nattokinase
*neo*^r^: kanamycin resistance gene
P_*gsiB*_: promoter of gsiB
P_*HpaII*_: promoter of HpaII
CDS: coding sequence
SP_*ap*_: the signal peptide of aminopeptidase
DO: dissolved oxygen
^mut^P_*srfA*_: the mutant of promoter P_*srfA*_
SLIC: sequence and ligation independent cloning

## References

[1] Terpe K (2006). Overview of bacterial expression systems for heterologous protein production: from molecular and biochemical fundamentals to commercial systems. Appl Microbiol Biotechnol.

[2] Vavrova L, Muchova K, Barak I (2010). Comparison of different *Bacillus subtilis* expression systems. Res Microbiol.

[3] Commichau FM, Alzinger A, Sande R, Bretzel W, Meyer FM, Chevreux B, Wyss M, Hohmann HP, Pragai Z (2014). Overexpression of a non-native deoxyxylulose-dependent vitamin B6 pathway in *Bacillus subtilis* for the production of pyridoxine. Metab Eng.

[4] Zafar M, Ahmed S, Khan MI, Jamil A (2014). Recombinant expression and characterization of a novel endoglucanase from *Bacillus subtilis* in *Escherichia coli*. Mol Biol Rep.

[5] Zaghloul TI, Abdelaziz A, Mostafa MH (1994). High level of expression and stability of the cloned alkaline protease (aprA) gene in *Bacillus subtilis*. Enzyme Microb Technol.

[6] Jung J, Yu KO, Ramzi AB, Choe SH, Kim SW, Han SO (2012). Improvement of surfactin production in *Bacillus subtilis* using synthetic wastewater by overexpression of specific extracellular signaling peptides, comX and phrC. Biotechnol Bioeng.

[7] Chen PT, Shaw JF, Chao YP, Ho THD, Yu SM (2010). Construction of chromosomally located T7 expression system for production of heterologous secreted proteins in *Bacillus subtilis*. J Agri Food Chem.

[8] Bhavsar AP, Zhao X, Brown ED (2001). Development and characterization of a xylose-dependent system for expression of cloned genes in *Bacillus subtilis*: conditional complementation of a teichoic acid mutant. Appl Environ Microbiol.

[9] Bongers RS, Veening JW, Van Wieringen M, Kuipers OP, Kleerebezem M (2005). Development and characterization of a subtilin-regulated expression system in *Bacillus subtilis*: strict control of gene expression by addition of subtilin. Appl Environ Microbiol.

[10] Nijland R, Lindner C, van Hartskamp M, Hamoen LW, Kuipers OP (2007). Heterologous production and secretion of clostridium perfringens beta-toxoid in closely related Gram-positive hosts. J Biotechnol.

[11] Kerovuo J, von Weymarn N, Povelainen M, Auer S, Miasnikov A (2000). A new efficient expression system for *Bacillus* and its application to production of recombinant phytase. Biotechnol Lett.

[12] Wenzel M, Muller A, Siemann-Herzberg M, Altenbuchner J (2011). Self-inducible *Bacillus subtilis* expression system for reliable and inexpensive protein production by high-cell-density fermentation. Appl Environ Microbiol.

[13] Trang TPP, Nguyen D, Schumann W (2012). Development of a strong intracellular expression system for *Bacillus subtilis* by optimizing promoter elements. J Biotechnol.

[14] Jan J, Valle F, Bolivar F, Merino E (2001). Construction of protein overproducer strains in *Bacillus subtilis* by an integrative approach. Appl Microbiol Biot.

[15] Lee SJ, Pan JG, Park SH, Choi SK (2010). Development of a stationary phase-specific autoinducible expression system in *Bacillus subtilis*. J Biotechnol.

[16] Li MJ, Wang JS, Geng YP, Li YK, Wang Q, Liang QF, Qi QS (2012). A strategy of gene overexpression based on tandem repetitive promoters in *Escherichia coli*. Microb Cell Fact.

[17] Kang HK, Jang JH, Shim JH, Park JT, Kim YW, Park KH (2010). Efficient constitutive expression of thermostable 4-alpha-glucanotransferase in *Bacillus subtilis* using dual promoters. World J Microb Biot.

[18] Guan CR, Cui WJ, Cheng JT, Zhou L, Guo JL, Hu X, Xiao GP, Zhou ZM (2015). Construction and development of an auto-regulatory gene expression system in *Bacillus subtilis*. Microb Cell Fact.

[19] Hamoen LW (2003). Controlling competence in *Bacillus subtilis*: shared use of regulators. Microbiology.

[20] Schneider KB, Palmer TM, Grossman AD (2002). Characterization of comQ and comX, two genes required for production of ComX pheromone in *Bacillus subtilis*. J Bacteriol.

[21] Roggiani M, Dubnau D (1993). ComA, a phosphorylated response regulator protein of *Bacillus subtilis*, binds to the promoter region of srfA. J Bacteriol.

[22] Phanaksri T, Luxananil P, Panyim S, Tirasophon W (2015). Synergism of regulatory elements in sigma(B)- and sigma(A)-dependent promoters enhances recombinant protein expression in *Bacillus subtilis*. J Biosci Bioeng.

[23] Gao X, Cui W, Tian Y, Zhou Z (2013). Over-expression, secretion, biochemical characterisation, and structure analysis of *Bacillus subtilis* aminopeptidase. J Sci Food Agric.

[24] Ben Khedher S, Zouari N, Messaddeq N, Schultz P, Jaoua S (2011). Overproduction of delta-endotoxins by sporeless *Bacillus thuringiensis* mutants obtained by nitrous acid mutagenesis. Curr Microbiol.

[25] Claverys JP, Prudhomme M, Martin B (2006). Induction of competence regulons as a general response to stress in gram-positive bacteria. Annu Rev Microbiol.

[26] Lopez D, Kolter R (2010). Extracellular signals that define distinct and coexisting cell fates in *Bacillus subtilis*. FEMS Microbiol Rev.

[27] Tyo KE, Ajikumar PK, Stephanopoulos G (2009). Stabilized gene duplication enables long-term selection-free heterologous pathway expression. Nat Biotechnol.

[28] Tyo KEJ, Ajikumar PK, Stephanopoulos G (2009). Stabilized gene duplication enables long-term selection-free heterologous pathway expression. Nat Biotechnol.

[29] Alper H, Fischer C, Nevoigt E, Stephanopoulos G (2005). Tuning genetic control through promoter engineering. Proc Natl Acad Sci USA.

[30] Bakke I, Berg L, Aune TE, Brautaset T, Sletta H, Tondervik A, Valla S (2009). Random mutagenesis of the PM promoter as a powerful strategy for improvement of recombinant-gene expression. Appl Environ Microbiol.

[31] Wu SM, Feng C, Zhong J, Huan LD (2011). Enhanced production of recombinant nattokinase in *Bacillus subtilis* by promoter optimization. World J Microb Biot.

[32] Phan TT, Nguyen HD, Schumann W (2012). Development of a strong intracellular expression system for *Bacillus subtilis* by optimizing promoter elements. J Biotechnol.

[33] Voskuil MI, Chambliss GH (1998). The −16 region of *Bacillus subtilis* and other gram-positive bacterial promoters. Nucleic Acids Res.

[34] Helmann JD (1995). Compilation and analysis of *Bacillus subtilis* sigma A-dependent promoter sequences: evidence for extended contact between RNA polymerase and upstream promoter DNA. Nucleic Acids Res.

[35] Haldenwang WG (1995). The sigma factors of *Bacillus subtilis*. Microbiol Rev.

[36] Cosby WM, Vollenbroich D, Lee OH, Zuber P (1998). Altered srf expression in *Bacillus subtilis* resulting from changes in culture pH is dependent on the Spo0K oligopeptide permease and the ComQX system of extracellular control. J Bacteriol.

[37] Li MZ, Elledge SJ (2007). Harnessing homologous recombination in vitro to generate recombinant DNA via SLIC. Nat Methods.

[38] Green MR, Sambrook J (2012). Molecular cloning: a laboratory manual.

[39] Bloor AE, Cranenburgh RM (2006). An efficient method of selectable marker gene excision by Xer recombination for gene replacement in bacterial chromosomes. Appl Environ Microbiol.

[40] Yan X, Yu HJ, Hong Q, Li SP (2008). Cre/lox system and PCR-based genome engineering in *Bacillus subtilis*. Appl Environ Microbiol.

[41] Miyazaki K, Takenouchi M (1033). Creating random mutagenesis libraries using megaprimer PCR of whole plasmid. Biotechniques.

[42] Botella E, Fogg M, Jules M, Piersma S, Doherty G, Hansen A, Denham EL, Le Chat L, Veiga P, Bailey K (2010). pBaSysBioll: an integrative plasmid generating gfp transcriptional fusions for high-throughput analysis of gene expression in *Bacillus subtilis*. Microbiology-Sgm.

[43] Gao X, Liu Z, Cui W, Zhou L, Tian Y, Zhou Z (2014). Enhanced thermal stability and hydrolytic ability of *Bacillus subtilis* aminopeptidase by removing the thermal sensitive domain in the non-catalytic region. PLoS One.

[44] Suwanmanon K, Hsieh PC (2014). Isolating *Bacillus subtilis* and optimizing its fermentative medium for GABA and nattokinase production. Cyta-J Food.

[45] Hartl B, Wehrl W, Wiegert T, Homuth G, Schumann W (2001). Development of a new integration site within the *Bacillus subtilis* chromosome and construction of compatible expression cassettes. J Bacteriol.

